# Electroconvulsive Therapy and Corpus Callosum Aplasia: A 3-Year Followup

**DOI:** 10.1155/2011/638506

**Published:** 2011-10-31

**Authors:** Ulrich Palm, Philipp Strauss, Christoph Born, Oliver Pogarell

**Affiliations:** Department of Psychiatry and Psychotherapy, Ludwig-Maximilian University, Nussbaumstraße 7, 80336 Munich, Germany

## Abstract

Electroconvulsive Therapy (ECT) is a powerful treatment option in severe or chronic catatonic states and has been reported to be useful in oligophrenic patients. We report the followup medical history of a patient with corpus callosum aplasia (or agenesis) who was continuously treated with ECT over three years. First, he improved considerably after a series of ECT, but relapses of catatonia made a continuous, weekly ECT necessary. Due to the severity of the brain malformation, an add-on medication with benzodiazepines and second generation antipsychotics was necessary to treat catatonic symptoms. This case emphasises the benefits of long-term ECT in oligophrenic patients.

## 1. Introduction

Recently, we reported the case of a 35-year-old male patient with isolated corpus callosum aplasia who underwent electroconvulsive therapy for a chronic catatonic state [1]. He was suffering from an organic catatonic and delusional disorder (DSM-IV criteria) for 15 years. His statomotoric and mental development was delayed, and cranial computer tomography revealed a callosal agenesis that was early linked to the developmental retardation. Upon the first admission to our hospital, the state of catatonia resolved during a series of eight right occipital ECT; afterwards, the patient was treated with quetiapine up to 1200 mg per day and lorazepam 3 mg per day. It was not possible to reduce this medication without re-exacerbation of catatonia; 3 mg of lorazepam was the threshold dose for muscle relaxing. EEG recordings during unilateral ECT revealed generalized seizure patterns most likely due to regular anterior and posterior commissural fibres, suggesting functional interhemispheric connectivity.

## 2. Case Report

Here, we report catamnestic information about treatment and course of disease in the last three years: after discharge from our hospital, the patient was referred to a special unit for adults with mental retardation. The medication of quetiapine 1200 mg and lorazepam 3 mg was continued, and right occipital ECT was performed weekly as maintenance treatment in order to prevent a relapse of catatonia. Several attempts to extend the ECT intervals (e.g., biweekly ECT) failed, since the patient showed severe deterioration at day 9 or 10 after the last ECT with increased muscle rigidity and mutism, followed by lack of motivation and movements. The symptoms resolved again after ECT and the treatment intervals were resumed to weekly intervals.

Severe catatonia relapsed once more when the patient was unable to ingest his medication due to pneumonia. Again, the patient was admitted to our hospital for another series of ECT. Although a right occipital ECT with a 65% energy set (337 millicoulombs) had formerly resolved the catatonia, a series of 3 treatments within one week showed no improvement at all. The treatment was extended to bilateral ECT, successively increasing energy to 130% (673 millicoulombs; current strength 0.92 A; frequency 70 Hz; pulse width 0.75 msec) within three treatments. These settings were tolerated well, and the catatonia subsided after another three treatments. Afterwards, bilateral ECT was continued weekly, providing stability over 10 months with adequate global functioning in the special service. 10 months later, despite regular ECT, the patient deteriorated within a few days, showing fearful affect, lack of impetus, and silent lip movements, suggesting hallucinations. These symptoms were similar to those reported upon his first admission to the hospital three years earlier [1]. Despite a series of another 8 ECT, mutism and a lack of impetus did not cease until the administration of additional medication with aripiprazole 10 mg per day. Nevertheless, ECT was continued, and the patient could be stabilized again by maintenance ECT every week.

## 3. Conclusions

Although most patients with mental retardation show severe and prolonged courses of disease, long-term outcomes are sparsely reported in the respective scientific literature [2–5], except for a paper by Tripp and Jacobson [6]. Here, the authors reported catamnestic data of a patient with FG (facial anomalies—gastrointestinal disorder) syndrome and continuous ECT over a timespan of three years [6]. Similarly, in our case, a severe malformation made continuous treatment necessary. The action of ECT in our case might be similar to the treatment of catatonic states in schizophrenia, leading to normalization of imbalanced neurotransmitter distribution. ECT has proven to be a safe and powerful tool in this case, leading to better results than the long-term medication alone in the past years. However, the severity of brain malformation made an additional psychopharmacotherapy necessary, for example, continuous treatment with lorazepam as a muscle relaxant or an augmentative therapy with quetiapine or aripiprazole. A combination of medication and ECT seemed to be more effective than a monotherapeutic approach. We suggest that ECT should be considered as an early therapeutic option and a long-term treatment even in psychiatric disorders due to brain malformation.
